# Elastic and inelastic strain in submicron-thick ZnO epilayers grown on *r*-sapphire substrates by metal–organic vapour phase deposition

**DOI:** 10.1107/S2052520624000441

**Published:** 2024-02-13

**Authors:** Maria Carmen Martinez-Tomas, Oleksii Klymov, Kazuki Shimazoe, Juan Francisco Sánchez-Royo, Mahesh Eledath Changarath, Said Agouram, Vicente Muñoz-Sanjosé

**Affiliations:** a Fisica Aplicada y Electromagnetismo, Universidad de Valencia, Dr. Moliner 50, Burjassot, Valencia 46100, Spain; b Department of Electronics, Kyoto Institute of Technology, Matsugasaki, Sakyo-Ku, Kyoto, 606-8585, Japan; c Instituto de Ciencia de Materiales de la Universidad de Valencia (ICMUV), 46980 Paterna, Spain; Moscow State University, Russian Federation

**Keywords:** high-resolution X-ray diffraction (HRXRD), thin films, unit-cell parameters, elastic strain, inelastic strain, critical thickness

## Abstract

A significant part of the present and future of optoelectronic devices lies on thin multilayer heterostructures. Their optical properties depend strongly on strain, being essential to the knowledge of the stress level to optimize the growth process. Here the structural and microstructural characteristics of sub-micron *a*-ZnO epilayers (12 to 770 nm) grown on *r*-sapphire by metal–organic chemical vapour deposition are studied. Morphological and structural studies have been made using scanning electron microscopy and high-resolution X-ray diffraction. Plastic unit-cell distortion and corresponding strain have been determined as a function of film thickness. A critical thickness has been observed as separating the non-elastic/elastic states with an experimental value of 150–200 nm. This behaviour has been confirmed from ultraviolet photoelectron spectroscopy, X-ray photoelectron spectroscopy and high-resolution transmission electron microscopy measurements. An equation that gives the balance of strains is proposed as an interesting method to experimentally determine this critical thickness. It is concluded that in the thinnest films an elongation of the Zn—O bond takes place and that the plastic strained ZnO films relax through nucleation of misfit dislocations, which is a consequence of three-dimensional surface morphology.

## Abstract 2

Sub-micron *a*-ZnO epilayers (12 to 770 nm) grown on *r*-sapphire by MOCVD have been analysed using SEM, HRXRD, UPS, XPS and HRTEM. The distortion of the unit cell has been determined and the consequent strain shows a transition from an inelastic to an elastic state, separated by a critical thickness; a method to experimentally determine this critical thickness is given.

## 1. Introduction

ZnO remains a material of high technological interest due to its applications in the field of optoelectronics, especially in the use of devices operating in blue and ultraviolet regions (Saeed *et al.*, 2023 ▸; Stefanovic *et al.*, 2023 ▸; Wang *et al.*, 2023 ▸). ZnO is a non-centrosymmetric crystal structure with a hexagonal lattice crystallizing in space group *P*6_3_ *mc* with unit-cell parameters *a* = 3.2470 Å, *c* = 5.2050 Å and γ = 120° (von Wenckstern *et al.*, 2012 ▸). The basis for the development of many of these optoelectronic devices is the growth of ZnO thin films and multilayers, which give rise, for example, to short-wavelength lasers, light-emitting diodes and high-density optical storage media (Kong, 2012 ▸; Pearton *et al.*, 2009 ▸).

One of the most common problems in the growth of thin films is to effectively control their structural and microstructural quality during their formation, knowledge of which allows for an adequate optimization process of growth parameters. Among the many factors affecting the properties of a thin film, strain is one of the most important. At the interfacial surface, defects and threading dislocations appear and can propagate through the epilayer (Ashrafi *et al.*, 2004 ▸; Yan *et al.*, 2015 ▸). These problems can influence electronic and optical properties, affecting both the device lifetime and operating wavelength (Abadias *et al.*, 2018 ▸; Hoat *et al.*, 2019 ▸). Consequently, an in-deep investigation of strain over a wide range on film thicknesses is needed.

Between different crystallographic orientations, ZnO layers with polar [c-(0001)] or semipolar [ 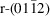 ] orientations exhibit built-in electric fields that worsen the electronic properties (Yan *et al.*, 2015 ▸). The undesirable spontaneous and piezoelectric polarization that appears in the active zone mainly affects the quantum efficiency of the devices based on these layers. Non-polar orientations, such as 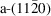 and 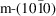 , can avoid these limitations (Jiu *et al.*, 2018 ▸). However, these last orientations, usually grown on *r*-sapphire and *m*-sapphire, respectively, are not exempt from problems. Sapphire is an excellent substrate, due to its high hardness, stable physical and chemical properties and good optical performance. Nevertheless, when growing *a*-ZnO on *r*-sapphire substrates, the large in-plane mismatch (1.55% along the *c*-axis and 18.3% along the *m*-axis) and different thermal coefficients lead to a distortion of the unit cell of ZnO that breaks the sixfold symmetry of the wurtzite structure. It is known that perturbations of the *C*_6*v*_ symmetry can seriously affect the optical properties of wurtzite materials and especially those of ZnO (Zúñiga-Pérez *et al.*, 2006 ▸).

Thus, a detailed knowledge of the microstructure and strain state of thin films seems to be desirable, if better understanding and control of multilayer-based devices are wanted.

In the current literature some studies dealing with the strain state of ZnO layers on sapphire can be found. Chauveau *et al.* studied the interface structure and anisotropic strain relaxation of nonpolar *a*-plane and *m*-plane ZnO (Chauveau *et al.*, 2008*a* ▸) and ZnMgO (Chauveau *et al.*, 2008*b* ▸) grown by molecular beam epitaxy (MBE) on sapphire. They worked with films of about 1 µm thickness. They found that the strong in-plane anisotropy results in different relaxation processes, depending on the in-plane direction. The main relaxation process is found to be through nucleation and glide dislocations. They found that low-energy prismatic slip systems provide an effective plastic relaxation because they are neither parallel nor perpendicular to the growth direction. Pant *et al.* (2010 ▸) investigated the anisotropic strain in nonpolar *a*-plane ZnO films grown on *r*-plane sapphire by detailed X-ray diffraction and high-resolution transmission electron microscopy (HRTEM) cross-section measurements. They grew films of ≃175 nm thickness and described the lattice distortion as a triaxially strained hexagonal structure, with a distorted basal plane in which the *a*-unit-cell parameter has different values along the in-plane *m*-axis direction of ZnO and the out-of-plane *a*-direction. Their HRTEM investigation of the interface shows that plastic relaxation by dislocation nucleation occurs at the interface surface.

There are also several studies on nitride compounds. Laskar *et al.* (2011 ▸) studied the distortion of wurtzite unit cells in nonpolar *a*-plane AlGaN films grown by metal–organic vapour phase epitaxy (MOVPE) on *r*-sapphire. They outlined a procedure to obtain unit-cell parameters and anisotropic strain, from which they derived an approximate expression to determine the Al content as solid phase; the studied epilayers were 0.8 µm thick. Jiu *et al.* (2018 ▸) investiged nonpolar GaN on patterned templates on sapphire. They obtained a reduction of the dislocation density by means of the overgrowth of GaN on regularly arrayed micro-rod templates on sapphire. Using this technique, they obtained a highly isotropic in-plane strain distribution and a low full width at half-maximum (FWHM) of the XRD rocking curve. Markurt *et al.* (2018 ▸) investigated plastic relaxation of GaN/Al_*x*_Ga_1–*x*_N_layer/GaN heterostructures grown by MOVPE on Si (111), sapphire (0001) and GaN (0001) substrates. They found that plastic relaxation proceeds mainly by nucleation and glide of *a*-type misfit dislocations in a slip-system driven by a three-dimensional surface morphology.

However, there are few studies of strain as a function of film thickness. Ashrafi *et al.* (2004 ▸) studied the thickness-dependent strain relaxation and its role on exciton resonance energies of epitaxial *c*-ZnO layers grown on 6H-SiC substrates. They determined the magnitudes of strain on these layers for thicknesses from 3 nm to 1.5 µm. Saraf *et al.* (2008 ▸) investigated the unit-cell deformation of *a*-plane ZnO films grown by metal–organic chemical vapour deposition (MOCVD) on *r*-sapphire, but they only go down to films of 355 nm thickness. They found that the unit-cell deformation is due to interfacial strain in films which in turn produces an in-plane anisotropic strain along the *c*-axis and *a*-axis directions of ZnO. Seo *et al.* (2014 ▸) analysed the effects of thickness on the electrical, optical, structural and morphological properties of Al and Ga co-doped ZnO films (AGZO) grown by linear facing target sputtering (LFTS). They found that these properties were critically influenced by the film thickness. The origin of this dependence was the change of the growth mode with increasing thickness, which came to affect the microstructure and surface morphology.

The goal of this paper has been to examine the structural and strain characteristics of *a*-ZnO films grown on *r*-sapphire substrates as a function of a wide range of film thicknesses, looking to establish the type of dependence. Thus by using high-resolution X-ray diffraction (HRXRD), the cell distortion and the consequent in-plane and out-of-plane strain of *a*-oriented ZnO epilayers grown by MOCVD on *r*-sapphire substrates have been analysed. The study has been made in the range of submicron thicknesses, covering the range from 12 to 770 nm.

The morphological and structural studies have been developed using scanning electron microscopy (SEM) and HRXRD, respectively. This study has been complemented by measurements of X-ray photoelectron spectroscopy (XPS), ultraviolet photoelectron spectroscopy (UPS) and high-resolution transmission electron microscopy (HRTEM), in order to assess the conclusions from the SEM and X-ray diffraction measurements.

## 2. Experimental

Thin ZnO films were grown at 375°C on *r*-plane sapphire substrates using MOCVD at atmospheric pressure in a horizontal Vent-Run-type quartz reactor (Quantax 226). Tert-butanol [(CH_3_)_3_COH] and di­ethyl zinc [(C_2_H_5_)_2_Zn] were used as oxygen (VI) and zinc (II) precursors, respectively, in ratio VI:II = 5. The flux parameters of oxygen and zinc precursors were 14.4 µmol min^−1^ and 2.9 µmol min^−1^, respectively. They were transported from temperature-controlled stainless steel bubblers using nitro­gen as the carrier gas. Pipelines were slightly heated to avoid condensation of precursors. Metal–organic and oxygen precursors had a separate inlet to the reactor, so the mixing and growth of the films took place directly on the heated substrate in the reaction chamber. The films thickness and surface morphology were controlled by increasing the growth time from 5 to 360 min in the different experiments.

The morphology and thickness of the samples were studied using a focused ion beam (FIB) field emission scanning electron microscope (FESEM) (SCIOS 2 FIB-SEM). A gold–palladium (4:1) thin conductive film (∼2 nm) was deposited over the ZnO films using dc-sputtering prior to the scanning analysis, in order to avoid the accumulation of electron charge on the layer and for increasing the signal-to-noise ratio. The samples were placed in a conductive aluminium holder specially designed for the simultaneous placement of various samples both horizontally and vertically.

HRXRD measurements were performed in a Panalytical X-Pert MRD diffractometer with a Cu tube. Parallel Cu *K*α1 irradiation was ensured by a parabolic mirror and a four-bounce hybrid monochromator situated in the incident beam. In the diffracted beam a gas-filled proportional detector and a fixed receiving slit of 1 mm were used. The X-ray beam divergences were 0.005° in the incidence plane and 2° in the axial direction. For the structural characterization of the samples, 2θ–ω scans and pole figures were carried out.

XPS and UPS measurements were performed in a SPECS GmbH system (base pressure 1.0 × 10^−10^ mbar) equipped with an ASTRAIOS 190 2D-CMOS hemispherical analyser. For the XPS measurements, photoelectrons were excited with the Al *K*α line (1486.7 eV) of a monochromatic X-ray source [μFOCUS 500 (SPECS GmbH)]. XPS measurements were taken at room temperature and normal emission with a pass energy of 20 eV. The size of the examined area was 1 mm × 1 mm. During measurements, pressure was under 7 × 10^−10^ mbar. For UPS measurements, photoelectrons were excited with the He-I line (21.22 eV) of a UVS µ-spot 300 UV light source; they were taken at room temperature and normal emission with a pass energy of 5 eV. For these measurements, the size of the examined area was 0.5 mm × 0.5 mm and the pressure was ∼5 × 10^−5^ mbar.

Energy-dispersive X-ray (EDX) analysis and HRTEM images were recorded with a Tecnai G2 F20 field emission gun transmission electron microscope under an acceleration voltage of 200 kV. For TEM measurements, ZnO films were scratched and deposited on a carbon-coated TEM copper grid.

## 3. Results

### 3.1. SEM measurements

**Figure 1:**
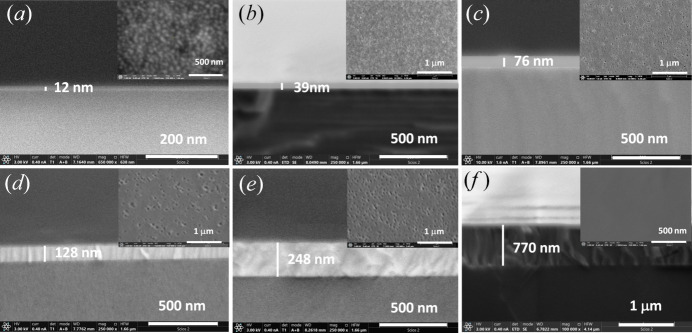
Representative surface and cross-sectional SEM images of ZnO films with different growth times: (*a*) 5 min 38 s, (*b*) 11 min 15 s, (*c*) 22 min 30 s, (*d*) 45 min, (*e*) 90 min, (*f*) 360 min

SEM measurements were performed to analyze surface and cross-sectional images of ZnO thin films (Fig. 1 ▸) obtained with different growth times.

The surface morphology can be observed in the insets of Figs. 1 ▸(*a*)–1 ▸(*f*), in which the films exhibit a smooth surface with the presence of some pinholes. Pinholes are small voids or craters that appear on the surface films. In the case of the thinnest film, grown with the shorter growth time [inset in Fig. 1 ▸(*a*)], a grain morphology has been observed. For films grown for a slightly longer time [Fig. 1 ▸(*b*)], the grain extends but pinholes appear. A random incomplete coalescence of grain can explain the presence of these voids (Irvine *et al.*, 2005 ▸).

For intermediate films [insets in Figs. 1 ▸(*c*), 1 ▸(*d*) and 1 ▸(*e*)] pinholes remain with more or less the same diameter and density (about 40–50 µm) and disappear for thicker films due to the continued overgrowth. The pinhole density indicates that the quantity of non-coalesced grains remains constant. This partial coalescence can be due to the use of a relatively low growth temperature. This consideration has been made by other authors (Xie *et al.*, 1999 ▸) who found that in GaN films grown by MOCVD, the density of random pinholes decreases by increasing the growth temperature.

Finally, for the thickest film [inset in Fig. 1 ▸(*f*)], a full recovery takes place resulting in a smooth and continuous surface, both in frontal and cross-section view.

**Figure 2:**
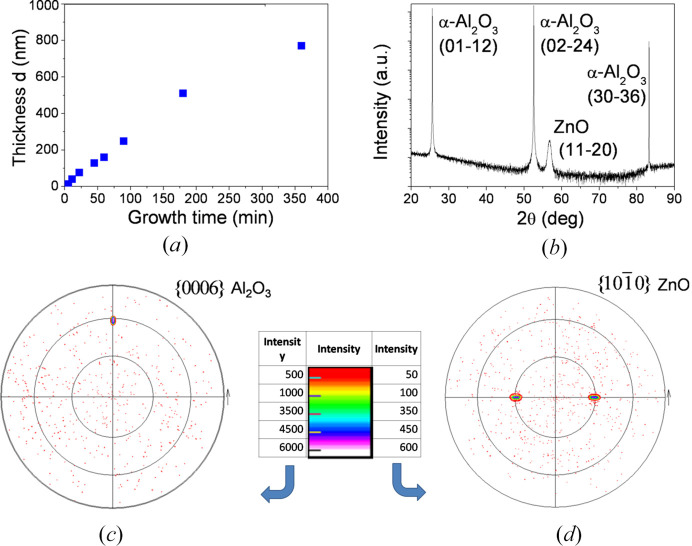
(*a*) Thickness of ZnO thin films as a function of growth time. (*b*) 2θ–ω scan of the 39 nm-thick ZnO film. Pole figures for (*c*) 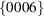 reflections of sapphire and (*d*) 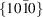 reflections of the 39 nm-thick ZnO film.

The thickness of layers was determined by directly measuring it in cross-section images [Figs. 1 ▸(*a*)–1(*f*)]. The thickness of films extends from 12 nm to 770 nm; Fig. 2 ▸(*a*) shows this dependence as a function of the growth time. The lateral views show that all films were homogeneous and with a progression towards a columnar structure, whose characteristics could be assessed by XRD measurements, as will be seen in Section 3.3.

### 3.2. Crystalline structure

The crystalline structure of ZnO samples was determined from 2θ–ω scans. Fig. 2 ▸(*b*) shows the diffraction pattern for the ZnO film of 39 nm thickness. This pattern is representative of all the samples.

Analysis of peak positions reveals that only two phases are present, that of sapphire (JCPDS card No. 00-010-0173) and that of ZnO (JCPDS card No. 00-036-1451) (JCPDS is Joint Committee on Powder Diffraction Standards). No other peaks can be seen in the patterns under the detection limit of XRD. The deposited ZnO films exhibit the 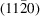 peak indicating that the ZnO films have a preferred *a*-orientation, with its *c*-axis parallel to the substrate surface. The 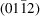 , 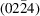 and 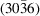 peaks of the *r*-sapphire substrate are also observed. We can then conclude that the out-of-plane orientation of ZnO 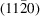 is parallel to the out-of-plane orientation of the *r*-sapphire substrate, namely Al_2_O_3_ 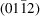 .

The in-plane orientation of films can be assessed by X-ray pole figures. Pole figures allow the determination of epitaxial relationships between film and substrate because X-rays penetrate through the ZnO film and into the Al_2_O_3_ substrate. Pole figures of the 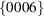 reflections of sapphire and of the 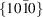 reflections of ZnO, for the 39 nm-thick film, can be seen in Figs. 2 ▸(*c*) and 2 ▸(*d*), respectively. The pole figure for sapphire shows the expected spot at a tilt angle of 57.61°. Poles of the 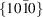 reflections for ZnO appear separated azimuthally by ±90° with respect to it and with a tilt angle of approximately 30°. This tilt angle is not the same for each sample, since slight variations due to the crystal deformation make this angle decrease from that of an undistorted value (30°) towards a lower angle (about 29.6°). Thus, these pole figures are representative of all others, keeping in mind that the poles are observed at approximately the same inclination angles.

This analysis allowed us to determine the epitaxial relationships between the *a*-ZnO layer and the *r*-sapphire substrate, which are


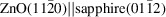
 (out-of-plane direction)


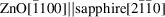
 (*m*-axis of ZnO direction)


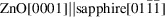
 (*c*-axis of ZnO direction)

**Figure 3:**
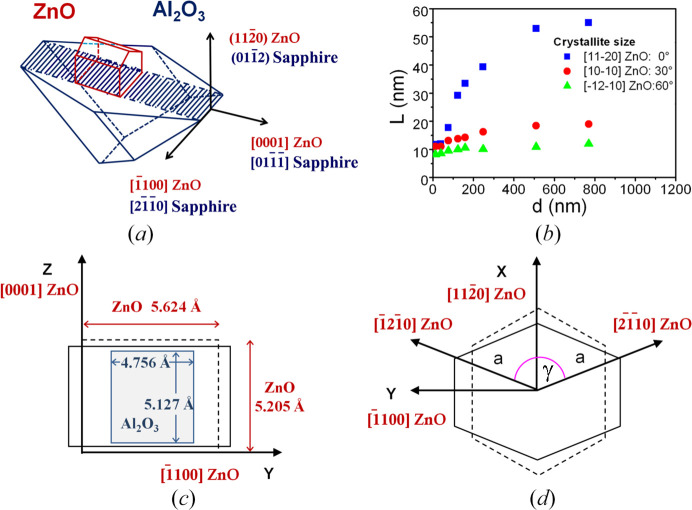
(*a*) Epitaxial relationships between *a*-ZnO film and *r*-sapphire substrate. (*b*) Calculated crystallite size as a function of the layer thickness for different inclination angles, at the φ angle of the *m*-axis. (*c*) Surface unit cell: sapphire (grey rectangle), undistorted ZnO (dotted line), distorted ZnO (solid line); indicated dimensions correspond to undistorted cells but lines are not to scale. (*d*) ZnO basal plane: undistorted ZnO with a perfect hexagonal shape (dotted line), distorted ZnO (solid line).

A scheme of these relationships can be seen in Fig. 3 ▸(*a*). This result reproduces the usual epitaxial behaviour of ZnO on *r*-sapphire substrates previously reported (Zúñiga-Pérez *et al.*, 2006 ▸; Pant *et al.*, 2010 ▸; Saraf *et al.*, 2008 ▸).

We can conclude from XRD measurements that the ZnO films, in the thickness range of 12 to 770 nm, grow epitaxially on *r*-sapphire substrates, with a well defined out-of-plane and in-plane orientation.

### 3.3. Microstructure

Layers grown on different substrates usually develop a sub-micrometre structure consisting of mosaic blocks constituted of coherent domains known as crystallites. Strain (understood as a change in the unit-cell parameters) primarily affects the shifting of diffraction peaks. However, microstrain (understood as a statistical displacement of atoms about their normal structural positions) and the finite size of crystallites contribute to the broadening of 2θ–ω diffraction peaks, among other broadening factors such as instrumental characteristics.

If microstrain effects are not considered, the size of crystallites can be calculated from Scherrer’s formula (Scherrer, 1918 ▸)

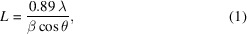

where *L* is the length of the coherent domain in the diffraction vector direction, λ is the wavelength of X-rays, β is the FWHM of the diffraction peak and θ is the Bragg diffraction angle.

It has to be noted that the Scherrer formula can only be applied for average sizes up to about 100–200 nm (Scherrer, 1918 ▸; Holzwarth & Gibson, 2011 ▸) and completely neglects the influence of lattice strain. Thus, in this study, the Scherrer formula has to be considered as an approximation to the crystallite size. Strain will be analysed in the following paragraphs.

As the Scherrer formula gives the size of crystallites in the direction of the diffraction vector, we have performed several 2θ–ω scans using skew-symmetric geometry in order to obtain the crystallite size for different inclination angles. Fig. 3 ▸(*b*) shows the calculated crystallite size of *a*-ZnO films obtained from the width of 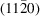 , 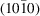 and 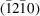 peaks, as a function of film thickness, after instrumental correction. Measurements were made at the φ angle of the *m*-axis. Thus the size obtained from the 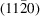 peak corresponds to the out-of-plane direction, the size obtained from the 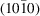 peak corresponds to an inclination angle of 30° from the out-of-plane direction and that from the 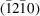 peak to an inclination angle of 60°. It can be seen that the crystallite size in the out-of-plane direction increases from 12 to 55 nm as the thickness was increased, while in the direction inclined at 60°, the crystallite size ranges from 9 to 12 nm. In both cases the last value corresponds approximately to the asymptotic value observed in Fig. 3 ▸(*b*). Calculations made at the φ angle of the *c*-axis give similar values. We can conclude that, at the beginning of growth, crystallites have a more or less spherical shape, but as the films become thicker, the growth along the out-of-plane direction is favoured over the transversal growth, giving rise to a columnar morphology. The aspect ratio for thick samples reaches a value of about 1:5. The increase in crystallite size can be interpreted as a consequence of the coalescence of small crystallites during the increase of the time of deposition. Specifically, initial nucleation points evolve towards small spherical crystallites and next to the bigger elongated ones, until the formation of a continuous film, as observed from SEM measurements.

### 3.4. Lattice distortion

The *a*-ZnO films grown on *r*-sapphire substrates exhibit anisotropic in-plane strain, unlike *c*-ZnO films grown on *c*-sapphire, which present isotropic strain. The origin of this anisotropy originates from the anisotropic in-plane lattice mismatch and from the different expansion thermal coefficients between layer and substrate.

Film–substrate mismatch appears because the in-plane parameters of *a*-ZnO are larger than those of *r*-sapphire (Chauveau *et al.*, 2018*b* ▸) [see dotted lines for ZnO and grey lines for sapphire in Fig. 3 ▸(*c*)], inducing an initial compressive state of ZnO in both directions. Specifically, the initial lattice mismatch is 1.55% along the ZnO *c*-axis, whereas it is as large as 18.3% along the *m*-axis, in the 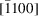 direction, as can be deduced from Fig. 3 ▸(*c*), where dimensions of both surface cells are given (Zúñiga-Pérez *et al.*, 2006 ▸). As we will see later, finally an in-plane biaxial anisotropic strain takes place [see solid lines in Fig. 3 ▸(*c*) for the distorted final surface cell of ZnO]. Thermal expansion coefficients also contribute to this anisotropy, as they differ both in the directions perpendicular and parallel to the *c*-axis [at 300 K: α⊥(ZnO) = 4.31 × 10^−6^ K^−1^, α∥(ZnO) = 2.49 × 10^−6^ K^−1^ (Iwanaga *et al.*, 2000 ▸), α⊥(Al_2_O_3_) = 6.2 × 10^−6^ K^−1^, α∥(Al_2_O_3_) = 7.07 × 10^−6^ K^−1^] (Lucht *et al.*, 2003 ▸). This combined anisotropy also leads to an additional deformation of the hexagonal basal plane of ZnO, giving rise to an overall orthorhombic distortion as can be seen in Fig. 3 ▸(*d*), where dotted lines are for the undistorted basal plane and solid lines are for the distorted basal plane].

To describe the orthorhombic lattice deformation induced on *a*-plane ZnO films grown on *r*-sapphire, we shall refer all distortions to the set of orthogonal reference axes shown in Fig. 3 ▸(*a*). These axes are the ZnO out-of-plane direction 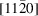 (*a*-axis) and the two ZnO in-plane directions 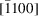 (*m*-axis) and 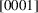 (*c*-axis). The periodicities of the unit cell labelled along the *a*-axis as 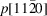 , along the *m*-axis as 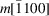 and along the *c*-axis as *c*[0001]; see also Figs. 3 ▸(*c*) and 3 ▸(*d*). The 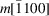 periodicity is twice the corresponding interplanar distance. In a perfect crystal, 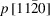 = *a* and *c*[0001] = *c*, but due to distortion and to the chosen angles [see Fig. 3 ▸(*d*)] these values are different from those indicated, as we will see next.

To determine the orthorhombic lattice deformation, we have followed the approach developed by Laskar *et al.* (2011 ▸) for accurately calculating the unit-cell parameters. They considered an orthorhombic distorted hexagonal system for which the interplanar distances are given by 

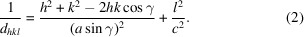

Fig. 3 ▸(*d*) shows the considered axes for the distorted basal plane, where the meaning of the *a* and γ parameters can be seen. It is noteworthy that the choice of the selected axes involves the reduction of the independent unit-cell parameters, because the interplanar distances along the 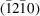 and 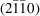 directions are the same and thus this expression reduces to that of a perfect hexagonal system by making γ = 120°.

Then, this expression has only three independent variables, namely *a*, *c* and γ, which can be evaluated by measuring the 2θ values of only three reflections of non-equivalent planes [for example 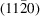 , 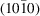 and 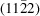 ]. However, to improve the accuracy of measurements it is better to measure several reflections.

Obtaining several reflections from very thin layers using a conventional high-resolution X-ray diffractometer is hard work. The main limitations are the reduced number of available reflections in the Ewald sphere and the impossibility of seeing simultaneously substrate peaks from asymmetric reflections. Another challenge is the lower intensity of the high-order reflections, which reduce the measurement precision.

We have performed several 2θ–ω scans using symmetric and skew-symmetric geometries. In order to overcome the previously mentioned drawbacks, we have measured the same reflection multiple times and using long acquisition times (up to several days), especially in the case of very thin films. As such, the uncertainty of measurements has been obtained in the interval from ± 0.0008 Å to ± 0.0014 Å. In Table 1 ▸, the indicated values in each column give the representative mean uncertainty for each parameter. From these measurements and the Bragg condition, interplanar distances were estimated and then averaged. The considered planes were the symmetric 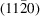 plane, the asymmetric 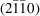 , 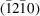 planes, and the asymmetric 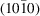 , 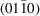 planes. Using equation (2) and via a standard least-squares error-minimization routine, the *a* and γ unit-cell parameters were obtained. Further, we next chose a set of axisymmetric planes which involve the *c* unit-cell parameter, such as 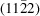 , 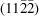 and 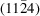 , 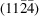 . Using equation (2) and the values previously determined for the *a* and γ unit-cell parameters, the value of *c* was determined, also using a least-squares error-minimization routine.

**Figure 4:**
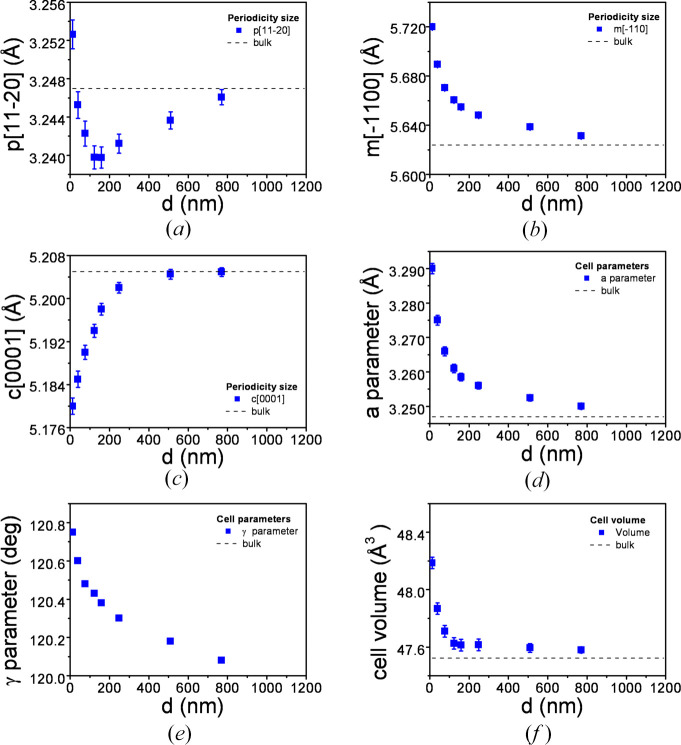
Evolution of periodicities along the orthorhombic axes as a function of film thickness: (*a*) out-of-plane ZnO 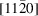 , (*b*) in-plane ZnO 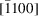 and (*c*) in-plane 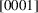 directions; (*d*) *a* and (*e*) γ unit-cell parameters; (*f*) unit-cell volume. Particular uncertainty has been indicated for each point; when error bars are not seen, it is because uncertainty is lower than the size of the symbol.

Periodicities 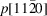 , 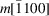 , 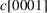 , and *a* and γ basal unit-cell parameters, obtained from these calculated values, are given in Table 1 ▸ and Fig. 4 ▸, as a function of film thickness. The unit-cell parameters for bulk ZnO have been taken as *a* = 3.2470 Å, *c* = 5.2050 Å and γ = 120° (von Wenckstern *et al.*, 2012 ▸; Hanada, 2009 ▸; Jang & Chichibu, 2012 ▸). The horizontal dashed lines in Fig. 4 ▸ correspond to these bulk values.

Calculations show that, as a general trend, the unit cell of ZnO presents the orthorhombic deformation described in Fig. 3 ▸(*d*). As a function of film thickness, the out-of-plane 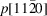 periodicity [Fig. 4 ▸(*a*)] has a changing behaviour. First it decreases, reaching a compressive state with a marked minimum, and then it increases tending to the bulk value. The distortion of the out-of-plane periodicity for the film of 12 nm is observed as tensile, but this behaviour might be an artefact because in films with a thickness below 30 nm, the unit-cell parameters of ZnO can be slightly different to those of the bulk value (Kalita & Kalita, 2015 ▸). The maximum absolute value for the compressive deformation takes place at a film thickness of about 150–200 nm.

The in-plane periodicity 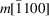 [Fig. 4 ▸(*b*)] is larger than the corresponding bulk value for all films, so it is under tensile stress. The other in-plane periodicity, *c*[0001] [Fig. 4 ▸(*c*)], is shorter than the corresponding bulk value for all films, so it is under compressive strain. Specifically, the in-plane strain has marked anisotropic biaxial characteristics.

The γ- and *a*-parameters [Figs. 4 ▸(*d*) and 4 ▸(*e*)] both have larger values than those of the bulk in all cases, this indicates that the *a*-parameter is under tensile stress.

The unit-cell volume [Fig. 4 ▸(*f*)] has been found to have a value greater than that of the bulk one, but only very thin layers (< 100 nm). For a film thickness of about 150–200 nm, the unit-cell volume is considerably reduced; then it tends smoothly to the bulk value. This type of dependence has been also found by other authors such as Wu *et al.* (2017 ▸) and Salluzzo *et al.* (2002 ▸), but only for layers of about several µm thickness.

This overall behaviour indicates that, in the very thin layer of 12 nm, both in-plane periodicities are highly strained, but the out-of-plane periodicity remains nearly undistorted. In addition, the unit-cell volume is much larger than that of the bulk volume, as a consequence of the dominant distortion of the 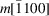 periodicity. For thicker films, as the relaxation process progresses, distortion of the out-of-plane periodicity increases, reaching a maximum absolute value of compressive strain for a film thickness of about 150–200 nm. From this point, the unit-cell volume tends quickly to the bulk value. The periodicities along the other axes also tend to bulk values for films with a thickness greater than 150–200 nm.

### 3.5. Strain

Once the unit-cell parameters are obtained, the strain can be calculated. Considering the *x*-axis along 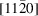 , *y*-axis along 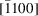 and the *z*-axis along [0001] directions [see Figs. 3 ▸(*c*) and 3 ▸(*d*)], the lattice deformation along the orthogonal axes will be evaluated against the bulk ZnO unit-cell parameters to calculate in-plane and out-of-plane strain components, according to equations

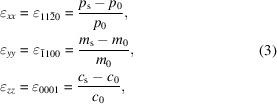

where the subindex 0 is for relaxed ZnO values and s is for strained values, ɛ_*xx*_ is the out-of-plane strain along 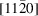 direction, ɛ_*yy*_ and ɛ_*zz*_ are the in-plane strain along 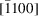 and [0001] directions, respectively.

**Figure 5:**
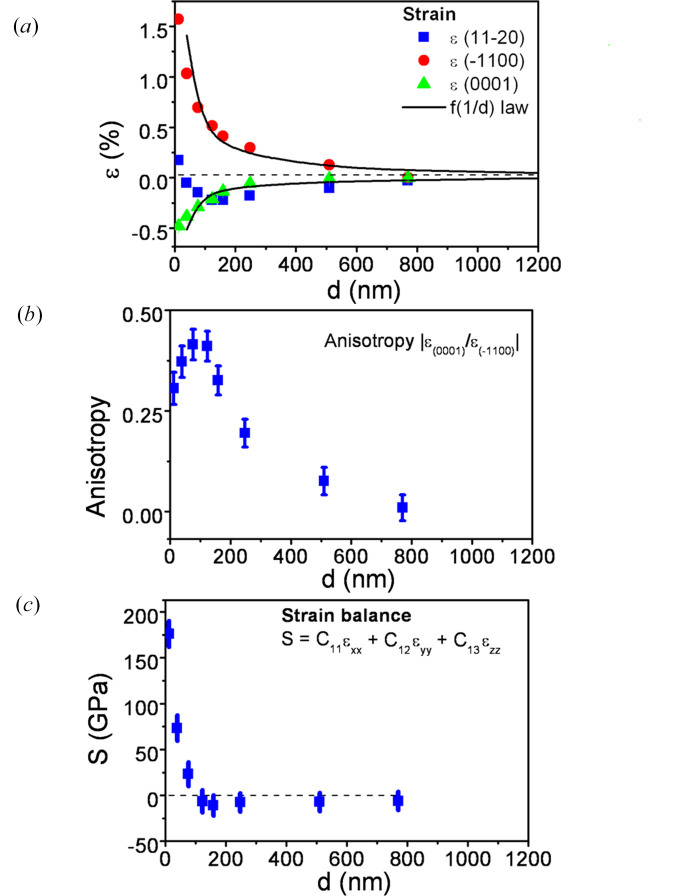
(*a*) Strain along the three orthogonal axes as a function of film thickness. (*b*) Anisotropy of the two in-plane strains, defined as the ratio between them. (*c*) Strain balance along the three orthogonal axes. Where error bars are not seen, it is because they are smaller than the size of the symbol.

Fig. 5 ▸(*a*) shows a plot of strain along the three orthogonal axes as a function of film thickness. This plot confirms the tensile strain along the 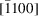 direction and the compressive strain along the 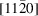 and [0001] directions. The highest strain takes place along the 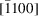 direction, consistent with the larger in-plane mismatch.

As the two in-plane strains are anisotropic, we have evaluated this anisotropy as the ratio between them in the form 
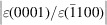
 . Fig. 5 ▸(*b*) exhibits the behaviour of this anisotropy as a function of the film thickness. It is seen that the strain anisotropy has a wide maximum for films with a thickness of about 150–200 nm. From this thickness it decreases, tending to zero for thick layers.

Measured strain can be understood as an overall strain. We have considered that it is constituted by pure lattice mismatch strain and by thermal strain. To separate both contributions, we have used the experimental law proposed by Saraf *et al.* (2008 ▸) and Freund *et al.* (2004 ▸) for in-plane and out-of-plane strains as a function of film thickness

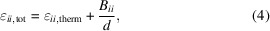

where ɛ_*ii*,tot_ (*i* = *x*,*y*,*z*), ɛ_*ii*,therm_ and *d* correspond to total strain, thermal strain and film thickness, respectively, while *B*_*ii*_ is a fitting coefficient.

The separation between lattice mismatch and thermal strain is made by fitting the experimental strain to this curve. It has been assumed that the strain components along the orthogonal axes are not interdependent and the *a*-ZnO film thickness is enough small to preserve a flat *r*-sapphire substrate, *i.e.* without curvature (Wietler *et al.*, 2006 ▸).

A dependence of strain which follows the *f* (1/*d*) law indicates a relaxation mechanism via forming misfit dislocations, in an agreement with the geometrical theory of strain relaxation (Dunstan *et al.*, 1991 ▸; Wang *et al.*, 2013 ▸). A fit of this law to the experimental data is shown in Fig. 5 ▸(*a*), thus indicating this type of relaxation mechanism.

Thermal strains and the calculated coefficients obtained from fitting to equation (4) are shown in Table 2 ▸. It is seen that thermal strain along 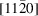 and [0001] directions is tensile, while along the 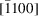 direction is compressive. In all cases thermal strain is a small portion of the total strain at room temperature.

### 3.6. Stress–strain tensor matrix

As well documented, stresses and strains are always present in thin films deposited on substrates. The relationship between them can be obtained in the framework of linear elastic theory by the stress–strain tensor, which completely defines the state of stress at a point inside a material in its deformed state. Using the axes of Figs. 3 ▸(*c*) and 3 ▸(*d*) (the *x*-axis along 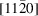 , *y*-axis along 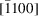 and *z*-axis along [0001] directions), the stress (σ_*ij*_) and strain (ɛ_*ij*_) relation for hexagonal crystals with a *C*6*v* symmetry can be expressed as (Laskar *et al.*, 2011 ▸; Grundmann & Zúñiga–Pérez, 2016 ▸)

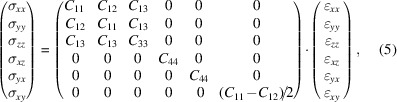

where *C*_*ij*_ are the stiffness constants and ɛ_*ij*_ are the strain components (*i*, *j* = *x*, *y*, *z*).

For an oriented *a*-plane hexagonal system as defined before, the crystal is free along the 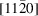 direction (or *x* axis), so σ_*xx*_ = 0 and then

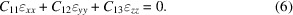

Specifically, based on linear elastic theory the sum of the strains along the three axes, weighted by the stiffness constants *C*_11_, *C*_12_ and *C*_13_, should be zero. The geometrical characteristics of this orientation make zero all shear strains (Laskar *et al.*, 2011 ▸; Mohuat & Coudert, 2014 ▸).

However, if we apply this equation to our results, the previous equation is not satisfied. Fig. 5 ▸(*c*) exhibits the sum *S* of three terms (*S* = *C*_11_ɛ_*xx*_ + *C*_12_ɛ_*yy*_ + *C*_13_ɛ_*zz*_) as a function of the film thickness. The values of the stiffness constants have been taken from Bateman (1962 ▸) as *C*_11_ = 209.7 GPa, *C*_12_ = 121.1 GPa, *C*_13_ = 105.1 GPa. It can be seen that for very thin films, at the beginning of growth, the result is far from zero, while for larger thicknesses it is close to zero. Note that we can consider valid the analysis of this balance even in the case of an orthorhombic geometry. The elastic matrix for ortho­rhombic systems has four additional independent elastic constants (namely, *C*_22_, *C*_23_, *C*_55_, *C*_66_), which does not affect equation (6). In addition, Dong & Alpay (2012 ▸) have calculated these constants for pure ZnO and for a ZnO supercell with orthorhombic structure, in the field of the density functional theory, finding that *C*_11_ ≃ *C*_22_, *C*_13_ ≃ *C*_23_, *C*_44_ ≃ *C*_55_, within a 5% difference. Then we can assume that these small differences in the stiffness constants of hexagonal/orthorhombic structures of ZnO cannot justify the great discrepancy in equation (6) for films with a thickness lower than 150–200 nm.

We can conclude then that strain in ZnO films cannot be interpreted in the framework of linear elastic theory along all the range of thicknesses. This interpretation is reinforced by the large volume of the unit cell for low thicknesses (in comparison to a relaxed cell), as observed in Fig. 4 ▸(*f*), and consequently in contrast to linear elastic theory. Thus, at the first stages of growth, we can assume that films are inelastically (*i.e.* nonlinearly) distorted, but for larger thickness they follow the theory of linear elasticity. The volume increasing of the unit cell for low thicknesses will be next confirmed and explained using XPS measurements through the expansion of the Zn—O bond.

In general, the inelastic relaxation process can be acquired by plasticity or fracture (Markurt *et al.*, 2018 ▸; Zhu & Li, 2010 ▸); however, neither cracks nor fractures were observed in our films. Salluzzo *et al.* (2002 ▸) found that when the mismatch becomes large, inelastic strain is more plausible. Li *et al.* (2014 ▸) have given values to this limit, reporting that traditional materials usually cannot sustain elastic strain exceeding 0.2–0.3%. In our case, this value is clearly exceeded by layers in the range of a ten of nanometres, for which strain clearly reaches values larger than the limit given before [see Fig. 5 ▸(*a*)].

A particular important concept to describe the structural characteristics of films is the critical thickness. From Fig. 5 ▸(*c*), we can see that there is an experimental critical thickness of about 150–200 nm for which the transition from an inelastic to an elastic behaviour takes place. This is in contrast to the theoretical critical thicknesses calculated according different models, as that of Hutchinson & Suo (1991 ▸) for cracking of tensile layers, or that of Holec *et al.* (2007 ▸) for plastic relaxation. These theoretical values generally are about 10–20 nm, far from our experimental value. However, experimental values for critical thickness in the hundreds of nanometres have been found by some authors for other materials or by using other growth techniques/conditions. For GaN, Markurt *et al.* (2018 ▸) obtained values in this range of thicknesses by developing a quantitative model for strain relaxation. Seo *et al.* (2014 ▸) investigated the effects of thickness on the electrical, optical, structural and morphological properties of Al and Ga co-doped ZnO films grown by LFTS; they found that below a critical thickness of 200 nm, the resistivity and optical transmittance of these films were significantly affected. As an interesting note, these authors also relate the critical thickness to the establishment of the stable columnar structure, as previously found from our SEM and XRD measurements.

Let us analyse the growth process within the critical thickness concept. Fig. 1 ▸(*a*) shows the surface SEM images of a ZnO film with a thickness of 12 nm; surface morphology is observed to be constituted by grains. Due to the anisotropic in-plane strain, these three-dimensional grains initially follow different in-plane growth modes. In the first stages, the growth along the *c*-axis of ZnO is via a pseudomorphic mode, due to the low mismatch in this direction (1.55%), following what is known as a lattice matching epitaxy (LME) model. In contrast, along the *m*-axis direction, the lattice mismatch is higher (18.3%) and consequently the strain relaxation requires extra-half planes of the sapphire substrates to reach the matching conditions. This growth mode is known as domain matching epitaxy (DME) model (Chauveau *et al.*, 2008*b* ▸). These different in-plane growth modes can be responsible for the fact that although the in-plane cell is initially in a compressive state, it finally reaches a tensile strain along the *m*-axis direction and a compressive strain along the *c*-axis direction. For films with a thickness of tens of nanometres [Figs. 1 ▸(*b*) and 1 ▸(*c*)], the morphology evolves to a coalescence state, as is usual in wurtzite structures. The partial coalescence of grains leads to big domains separated by voids. When thicknesses are in the order of hundreds of nanometres [Figs. 1 ▸(*d*) to 1 ▸(*f*)], the relaxation process leads to the formation of dislocations, as confirmed by the hyperbolic dependence of the strain and also observed by other authors (Wang *et al.*, 2013 ▸; Lee *et al.*, 2010 ▸). As strain is progressively relieved by dislocations, the local inelastically strained layers, grown at the prior stages of growth, lose significance and an average linear strain behaviour dominates.

For analysis of the critical thickness, elastic energy has to be considered. In the Matthews–Blakeslee critical thickness model (Matthews & Blakeslee, 1974 ▸; France *et al.*, 2015 ▸), the key point is the balance between the increase in elastic energy surrounding a dislocation and the decrease in misfit energy as a dislocation glides. In the in-plane growth along the *c*-axis direction, the elastic energy is high, because the slip systems necessary for strain relaxation are pyramidal slip systems (neither parallel nor perpendicular to the growth direction), which need a high energy to be activated. This leads to a high elastic energy, only relieved at high critical thickness, as we have previously found. In contrast, along the *m*-axis direction, the slip system needed for strain relaxation is a prismatic slip system (parallel to the growth direction), which requires a lower energy. So, in our case, the critical thickness is imposed by the pyramidal slip system.

With respect to the sudden behaviour in the relaxation process, Li *et al.* (2014 ▸) explained that the strain ɛ(*x*), which describes the Bravais lattice distortion at a given point *x*, is composed of elastic and inelastic strain. The elastic strain ɛ_e_(*x*) would be spatially delocalized and the inelastic strain ɛ_i_(*x*) spatially localized. These authors establish that there is an elastic limit ɛ_lim_ similar to the solubility limit of a single phase in chemical free energy. A strain ɛ beyond this limit would cause a ‘precipitation’ of the total strain into inelastic strain ɛ_i_(*x*). The abrupt change seen in Fig. 5 ▸(*c*) seems to experimentally confirm this behaviour, as the critical thickness at about 150–200nm is the point at which inelastic behaviour begins. Thus the sum *S* of the strains along the three axes, weighted by the stiffness constants, (*S* = *C*_11_ɛ_*xx*_ + *C*_12_ɛ_*yy*_ + *C*_13_ɛ_*zz*_), can be proposed as a useful method to determine the critical thickness. As seen in the Fig. 5 ▸(*c*), a value of *S* = 10 GPa is the limit that indicates if the film has reached an elastic behaviour.

The earlier HRXRD analysis has led us to assess, not only the known orthorhombic distortion of the cell unit in ZnO films grown on *r*-sapphire, but also its evolution as a function of the film thickness. We have determined the existence of a critical thickness, which is found to be in the 150–200 nm range of film thickness. In order to confirm these findings, additional XPS and HRTEM measurements were made.

### 3.7. XPS and UPS measurements

Electronic properties of the films as a function of film thickness have been studied by XPS and UPS techniques. With this aim, the edges of sapphire substrates were electrically grounded to a sample holder using silver paste. Additionally, we have adopted the procedures described by Greczynski & Hultman (2017 ▸, 2022 ▸) to achieve an accurate determination of the binding energy of electronic states of samples. In XPS and UPS measurements, no appreciable shift of the signal has been observed. Thus we can say that no eventual charging effect happens, under the detection limit of our apparatus.

**Figure 6:**
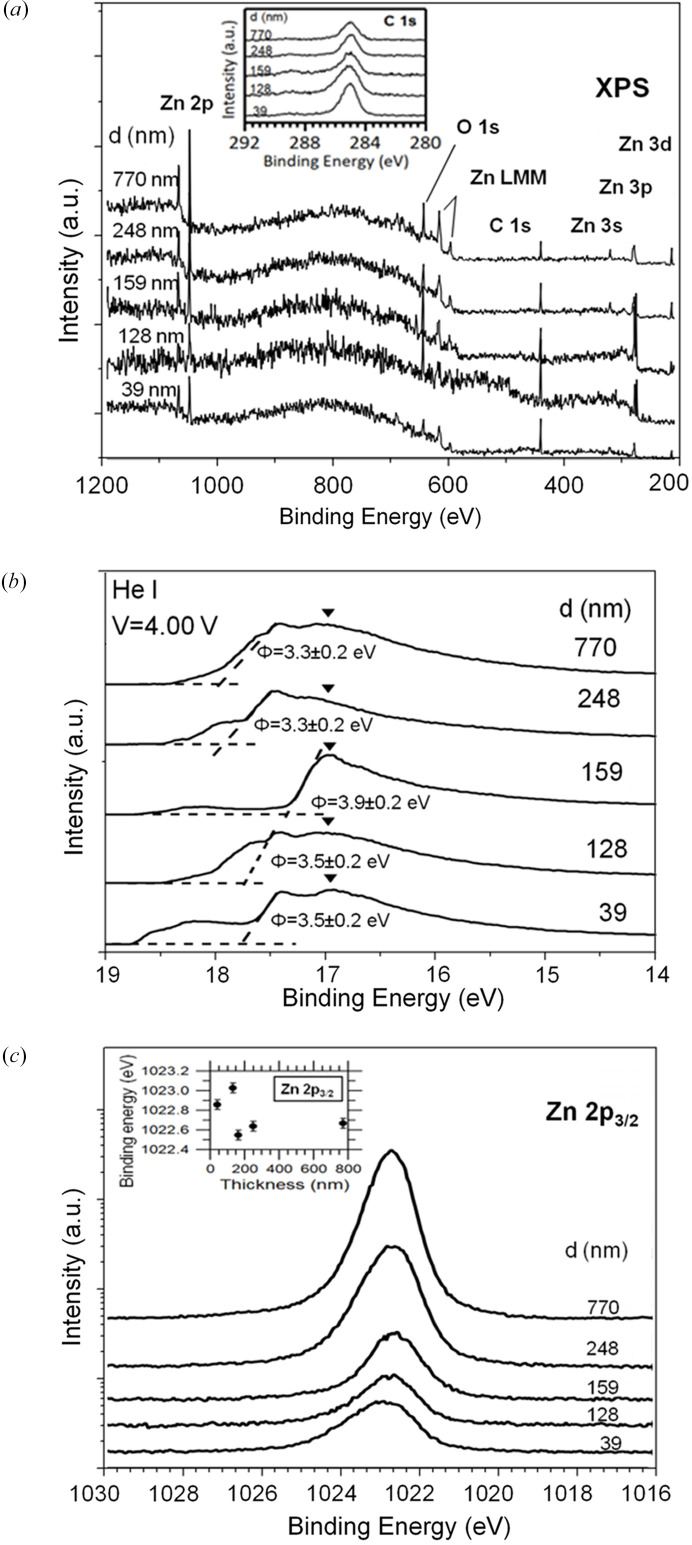
(*a*) XPS survey spectrum of samples. The C 1*s* spectra can be seen in an inset. (*b*) UPS spectra of samples. Inverted black triangles indicate the feature attributable to non-hydrated areas of each sample. Features appearing at higher binding energies have been attributed to hydrated areas of samples. The secondary photoemission cut-off preceding these features has been used to determine the work function of the hydrated samples by linear extrapolation, as illustrated by dashed lines. The obtained work function values have been indicated under each spectrum. (*c*) High-energy-resolution XPS spectra of the Zn 2*p*_3/2_ core level measured in ZnO films of different thickness. The inset shows the Zn 2*p*_3/2_ peak position as a function of thickness.

Fig. 6 ▸(*a*) shows the XPS survey spectra acquired in ZnO samples of different thickness, which reveal the additional presence of adventitious carbon at the surface of all samples. The C 1*s* signal coming from adventitious carbon is usually employed as a binding energy reference and their respective spectra are shown in the inset of Fig. 6 ▸(*a*). This procedure gives accurate results when the work function of each particular sample is known (Greczynski & Hultman, 2017 ▸, 2022 ▸).

Data shown in Fig. 6 ▸(*b*) show the UPS spectra acquired in samples when a gate voltage of *V* = −4.0 eV was applied during the photoemission measurements. By applying a negative gate voltage, the UPS spectrum shifts to lower binding energies, allowing the measurement of the secondary photoemission cut-off, which for most samples lie beyond the measurement window. The spectra shown in Fig. 6 ▸(*b*) have been already shifted back by subtracting the gate voltage value applied during the acquisition process. The secondary photoelectron cut-off measured in these samples reveals a complex structure at the high binding energy side of the spectra, probably due to a collateral effect of applying gate voltages to ZnO films which were grown on a dielectric substrate. This particular experimental configuration is also responsible for the fact that secondary cut-off is far from sharp and appears as smooth ramps, as indicated by the inclined dashed lines in Fig. 6 ▸(*b*). UPS results support the existence of a critical thickness of ZnO films at this interval. In the samples with a thickness other than 159 nm, two photoemission signal peaks can be observed, but in this particular sample, only one peak has been observed. The peaks observed at lower binding energies, indicated by inverted black triangles, seem to appear at similar energies in all samples, which suggests that this peak can be attributable to non-hydrated areas of the samples. Peaks observed at higher binding energies can be attributed to hydrated areas of the samples (Gutman *et al.*, 2012 ▸). As the secondary photoelectron cut-off of the hydrated area overlaps with that of the non-hydrated area, work function values have been calculated for the hydrated areas of the films by linear extrapolation of the secondary photoelectron cut-off [dashed lines in Fig. 6 ▸(*b*)]. Obtained values have been indicated on each spectrum. Films thinner than 159 nm have work function values of Φ = 3.5 eV. In the 159 nm-thick film, the work function sharply increases to reach a value as high as Φ = 3.9 eV, which is close to those found in other polycrystalline ZnO films (Φ = 3.7–4.3 eV) (Kuo *et al.*, 2012 ▸; Sharma *et al.*, 2013 ▸, 2016 ▸). However, films thicker than 159 nm present work function values even lower than those found in the thinner films. Pre-edge signals observed at binding energies higher than the secondary cut-off are attributed to photoelectrons originating in the sapphire substrate and accelerated by the gate potential applied. It has been reported that hy­droxy­lation of the ZnO surface tends to reduce its work function (Gutmann *et al.*, 2012 ▸). Also, morphological roughness seems to reduce the work function of ZnO films (Sharma *et al.*, 2016 ▸).

Fig. 6 ▸(*c*) shows the Zn 2*p*_3/2_ core-level spectra measured using XPS in ZnO films of different thickness. These are characterized by the presence of spin-orbit doublets whose spin-orbit splitting is 23.1 eV and their Zn 2*p*_3/2_ peaks are located around 1021.5 eV, in accordance with values observed by other authors for ZnO (Best *et al.*, 1977 ▸; Strohmeier, 1984 ▸). For each one of the samples measured, a single doublet has been clearly identified, which is attributable to the usual configuration of Zn in a Zn^2+^ oxidation state. These measurements may explain the large unit-cell volume found in the thinnest films. The measured increase of the positive on-site potential of the Zn^2+^ ions and of the binding energy Zn 2*p*_3/2_ core levels indicate an increase of the Zn—O bond length and an enhancement of the dipolar moment. Although the shift could also affect the binding of O 1*s* photoelectrons, we could not clearly identify them from our O 1*s* spectra. This is probably due to the presence of other O 1*s* components and to the fact that oxygen atoms, as they are very electronegative and mostly capture electrons from Zn atoms, are less sensitive to small bond-length variations. A detailed inspection of spectra acquired in films thinner than 128 nm reveals that core-level peaks tend to exhibit some asymmetric line shape [see Fig. 6 ▸(*c*)], which contrasts with the symmetric one of the thicker films. This asymmetry may be attributable to band bending effects on the ZnO side of the ZnO/sapphire heterointerface. Also, it can be observed that spectra measured in films thicker than 159 nm are downshifted by 0.5 (1) eV with respect to those recorded in thinner films [see inset in Fig. 6 ▸(*c*)], assessing the existence of a critical thickness in the range 150–200 nm.

From these findings, it appears that strain in the thinnest films produces the lengthening of bonds, characteristic of nanometric films, as referenced by other authors (Ennaceri *et al.*, 2019 ▸; Lyahovitskaya *et al.*, 2012 ▸; Vasconcelos Borges Pinho *et al.*, 2021 ▸). It is accepted that for nanoscaled materials, *i.e.* below 100 nm, the expansion of the unit cell begins to take place, and especially when the size reduces to 10 nm, as well for grains and nanowires, as for films. The effective volume of the unit cell is then coherently increased, according to Fig. 4 ▸(*f*).

These facts indicate that XPS and UPS results here reported are a consequence of the morphologic and structural properties of the ZnO films studied in this work, at least along the crystalline direction perpendicular to the film surface (*i.e.* the 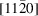 ), which seems to present a thickness-dependent behaviour characterized by a critical thickness located in the range 150–200 nm.

### 3.8. HRTEM measurements

**Figure 7:**
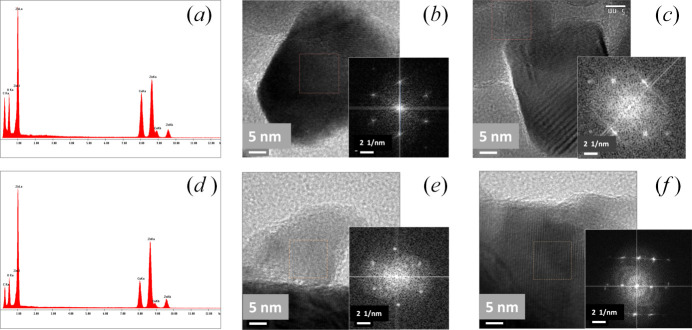
For a ZnO film with a thickness of 12 nm: (*a*) EDX spectrum, (*b*) HRTEM image of basal plane, (*c*) HRTEM image of a prismatic plane. For a ZnO film with a thickness of 770 nm: (*d*) EDX spectrum, (*e*) HRTEM image of basal plane, (*f*) HRTEM image of a prismatic plane. The insets are the FFT of the selected area in the HRTEM images.

Energy-dispersive X-ray (EDX) and HRTEM studies were performed to complement SEM, HRXRD, UPS and XPS analyses in order to assert the orthorhombic distortion of the wurtzite cell. A small quantity of material from samples with a small film thickness (12 nm) and with a large film thickness (770 nm) were scratched and deposited on copper gratings. Figs. 7 ▸(*a*) and 7 ▸(*d*) show the EDX images obtained from these films. Spectra consist of different Zn, O, Cu and C peaks in both samples. The Cu peak came from the Cu TEM grids and the C peak came from the carbon-coated copper grid. No other elements were found.

Figs. 7 ▸(*b*), 7 ▸(*c*), 7 ▸(*e*) and 7 ▸(*f*) show HRTEM images of the scratched material and the insets the corresponding fast Fourier transform (FFT). Defined (*hkil*) spots on the FFT images reflect the crystalline nature of the samples. In Figs. 7 ▸(*b*) and 7 ▸(*e*) spots correspond to the basal plane of a more or less hexagonal structure. For the sample with a thickness of 12 nm [Fig. 7 ▸(*b*)] the spots are distributed in an irregular hexagonal shape, while those of the sample with a thickness of 770 nm [Fig. 7 ▸(*e*)] present a regular distribution. In both cases spots can be assigned to the family of ZnO 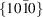 planes of a distorted/undistorted hexagonal structure. The experimental 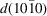 values from the irregular hexagonal symmetry give different values, depending on the selected direction in the irregular hexagon. The largest one is [3.01 (2) Å], which means that the *p*-periodicity [which is twice 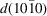 ] is *p* = 6.02 (4) Å, *i.e.* higher than ZnO bulk *p*-periodicity [*p* = 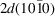 = 5.624 Å] as expected from Fig. 4 ▸(*b*). This distortion of the basal plane confirms the orthorhombic deformation of the wurtzite ZnO structure. Figs. 7 ▸(*c*) and 7 ▸(*f*) show the spots corresponding to prismatic planes, with spots that can be assigned to the 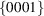 , 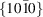 and 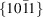 families. The obtained *d*_0001_ values (*i.e.* the *c*-periodicity) for the thinner film (12 nm) and for the thicker one (770 nm) were 5.18 (2) Å and 5.19 (2) Å, respectively. These values agree with Fig. 4 ▸(*c*), within the margin of uncertainty. The observed crystalline zones are within the interval of crystallite sizes determined from XRD measurements [Fig. 3 ▸(*b*)].

## 4. Summary and conclusions

An accurate analysis of the microstructure and strain relaxation has been made on *a*-oriented films of ZnO grown on *r*-sapphire by MOCVD with thicknesses in the wide sub­micron range 12–770 nm. The objective was to determine the structural characteristics and strain state as a function of film thickness. The study has been carried out by performing SEM, HRXRD, UPS, XPS and HRTEM measurements. Detailed X-ray scans have been made along symmetric and skew-symmetric directions and pole figures have been obtained. From these measurements, we have analysed the structural and elastic properties of ZnO epitaxial films as a function of film thickness. We have found that a critical thickness of about 150–200 nm marks a point of change below which important structural changes take place. For thin films, the calculated shape of crystallites is shown to be a more or less spherical, while thicker ones become columnar, beginning with the elongation of crystallites at this critical thickness and giving rise to the stable columnar structure. The lattice deformation has been found to be orthorhombic and the corresponding distortion has been calculated as a function of film thickness, using three orthogonal axes, more adequate for this type of distortion. The strain is found to be compressive along the 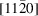 and [0001] directions (defined as the *a*-axis and the *c*-axis, respectively) while it is tensile along the 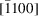 direction (defined as the *m*-axis). Considering the measured strain as an overall value, thermal strain has been separated from lattice mismatch strain by curve fitting, finding that thermal strain at room temperature is always a small portion of the total strain. The critical thickness mainly marks the transition from an inelastic relaxation process to an elastic one. Usually the inelastic relaxation process can be acquired by plasticity or fracture, but we have observed only a plastic behaviour in our samples, which presumably is responsible of the orthorhombic distortion of the unit cell. This deformation is also confirmed from HRTEM images. Indeed, in very thin films an enlarged unit-cell volume has been found, which has been explained by XPS as produced by an elongation of the Zn—O bond, typical of nanometric strained films. The plastically strained magnitudes have a marked distortion until the critical thickness, but beyond it, they evolve to an elastic behaviour, until a complete relaxation. This transition is especially abrupt for the balance of strains along the three axes. We propose that this balance can be used as an easy experimental way to determinate the critical thickness. The analysis of the SEM images, as well of the XRD results, indicate that the relaxation of plastic strained ZnO films proceeds predominantly by nucleation of misfit dislocations consequence of a three-dimensional surface morphology.

## Figures and Tables

**Table 1:** Periodicities (Å) 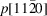 , 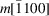 , c[0001] of the orthorhombic unit-cell and basal unit-cell parameters *a* (Å) and γ (°) for films with different thickness (nm) The indicated uncertainties give a representative mean value of them.

Film thickness (±3%)	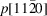 (± 0.0011)	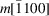 (± 0.0011)	*c*[0001] (± 0.0011)	*a*-parameter(± 0.0011)	γ-parameter (± 0.01)
12	3.2526	5.7199	5.1800	3.2900	120.75
39	3.2453	5.6895	5.1850	3.2750	120.60
76	3.2423	5.6705	5.1900	3.2660	120.48
128	3.2398	5.6604	5.1940	3.2610	120.43
159	3.2398	5.6547	5.1980	3.2585	120.38
248	3.2412	5.6481	5.2020	3.2560	120.30
510	3.2436	5.6386	5.2045	3.2525	120.18
770	3.2461	5.6314	5.2049	3.2500	120.08
Bulk	3.2470	5.6240	5.2050	3.2470	120.00

**Table 2:** Thermal strain and fitting coefficients as a function of film thickness, for the 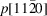 , 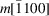 and c[0001] periodicities at room temperature

Direction	ɛ_*ii*,therm_ (%)	*B*_*ii*_ (nm)
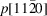	0.0041 (±0.0005)	−54.6 (±0.5)
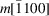	−0.0572 (±0.0005)	70.0 (±0.5)
*c*[0001]	0.0379 (±0.0005)	−26.4 (±0.5)
